# Real-World Experience on Why Research Flatlines: A Review of Trials From the Coordinator’s Perspective

**DOI:** 10.7759/cureus.51703

**Published:** 2024-01-05

**Authors:** Megan Koenig, Andrea Castro Cara, Anna Woods, Terrie Vasilopoulos, Amy M Gunnett

**Affiliations:** 1 Anesthesiology, University of Florida College of Medicine, Gainesville, USA

**Keywords:** unsuccessful clinical trials, inadequate study population, study design flaws, inadequate budget, clinical trial planning, staff turnover, poor enrollment, clinical research failure

## Abstract

Introduction: Investigator-initiated research trial failure is a national concern that hinders the dissemination of information while wasting resources, time, and funding. The goal of this analysis was to provide an objective review of points to consider increasing an investigator’s chances of success.

Methods: The included trials were divided into two groups based on whether they were successful or unsuccessful in meeting enrollment goals. Common issues were noted for each trial to identify prevalent issues and compare their quantity within each group.

Results: Unsuccessful trials averaged twice as many issues as trials in the successful group. The most common problems identified in unsuccessful studies involved study planning, whereas the most common problems identified in successful studies revolved around study staff.

Conclusions: There is no single definitive indicator for trial failure; however, awareness of these issues in a trial’s planning phase can help prevent their occurrence and aid in overall completion and publication.

## Introduction

Research trial failure is a real concern for academic research offices regarding both industry-sponsored and investigator-initiated trials. According to Butryn et al., “Clinical trial (CT) sites face a continually changing legal and regulatory landscape, making research conduct and program operations challenging” [1]. The Office of Research in the Department of Anesthesiology at the University of Florida College of Medicine currently oversees over 200 active trials in various stages. Investigator-initiated research makes up approximately 90% of those active trials, led by a team of approximately 80 investigators and seven study coordinators. Poorly designed, inadequately vetted, or mismanaged research projects pose an unnecessary and unreimbursed burden on administrative and clinical research staff. Many of these eventually become stalled, flatlined, or failed studies. For the purpose of this paper, research trial failure is defined as a study that fails to complete enrollment, especially within study-projected timelines. Problems resulting from inadequate evaluation of trial feasibility, logistics, finances, trial design, and statistical analysis contribute to the issues.

This problem is not unique. The struggle to reach enrollment goals in trials is a national concern, regardless of sponsorship. A 2018 survey of 245 trials registered on ClinicalTrials.gov in the US, UK, and Canada found that 56% of trials suspended, withdrawn, or terminated between 2006 and 2017 did not enroll any participants (Poster: Reynolds P. Why Academic Trials Fail: Trial 'Cemetery Demographics' and a Case Study of Early Termination). University of Florida, College of Medicine, Department of Anesthesiology, Celebration of Research. May 3, 2017. Among those that did enroll participants, the median enrollment was only 16% of the targeted enrollment. Reynolds further reported that, in this sample, over 4,000 patients were enrolled in trials that were never completed. In a similar study, Carlisle et al. reported that “of 2579 eligible trials, 481 (19%) either terminated for failed accrual or completed with less than 85% expected enrolment” [2]. Under-enrollment remains a vexing problem in clinical research, and efforts to address it are hampered by a limited understanding of its causes. While our findings do not provide a definitive explanation for this phenomenon, they suggest that enrolling human subjects in clinical research is subject to unique organizational and cultural factors that have not been well characterized or systematically studied.

Logistical errors may arise at any point between designing a study and disseminating information. Clinical trial budgets are often underfunded, especially those that are investigator-initiated. However, all studies require sufficient financial, personnel, and time resources to complete the necessary regulatory, institutional review board (IRB), and study startup requirements. Poorly designed studies can leave research questions unanswered. When a trial fails, the consequences extend beyond a lack of new information: resources are wasted, and translatable research findings are left dangling.

In 2011, only 78 of 150 studies registered with ClinicalTrials.gov published results within two years of completion. Therefore, roughly half of clinical trials remained unpublished for extensive periods of time despite the original intentions of contributing to the evidence base for practice [3]. This lack of dissemination means that results, positive or negative, cannot constructively impact future study applications, and it undermines the bioethical promise made to study volunteers. Butryn et al. stated, "Many trials fail to achieve their primary endpoints and often fall short of their secondary endpoints as well. The reasons for these failures are multifactorial and can be related to issues in trial design, patient selection, dosing, drug formulations, unexpected adverse events, and many other factors" [1].

To perform clinical research with rigor and reproducibility, as well as to improve the effectiveness of our research office, we performed an impact analysis of clinical studies. This analysis was designed as a first step toward understanding performance metrics and the factors that contributed to failed studies. We sought to compare successful studies to unsuccessful studies during our study period.

## Materials and methods

We performed a retrospective quality audit of all trials conducted through the Office of Research for the Department of Anesthesiology at the University of Florida College of Medicine that were actively enrolling trials in the previous calendar year. The team considered all of the department’s projects and had a round-table discussion about each actively enrolling study. The team identified common difficulties and barriers in relation to each active study and, during the discussion, identified additional barriers such as a lack of principal investigator commitment and poor communication. Because this was a quality assurance project and personal information was not gathered and reported, IRB review was not required. Twenty-three studies were reviewed, focusing on factors impacting study performance metrics. A total of 16 factors were identified (Table 1).

**Table 1:** Common research study factors identified for analysis

Protocol changes	Poor budgeting
Subjectivity in data collection	Failure to collect standard-of-care interventions
Staff turnover	Lack of principal investigator commitment
Design flaws	Poor communication
Poor staff training	Poor enrollment strategies
Subjects lost to follow-up	Inappropriate subject recruitment
Lack of trial population	Technical issues
Changing trial costs	Lack of clinical staff cooperation

Not all of these issues are independent. For example, design flaws may cause multiple or major protocol changes and poor budgeting, while a lack of principal investigator commitment can lead to poor communication that negatively affects the project.

After these issues were identified, we divided the sample into two groups: successful studies and unsuccessful studies. The positive outcome used to deem the study “successful” was having recruited the intended number of subjects in the original estimated time frame, whereas the negative outcome that deemed the study “unsuccessful” was failing to achieve the desired trial population in the original estimated time frame. Within each group, we identified logistical errors in trial design and execution to assess root cause factors for the unsuccessful results. The trials encompassed many types of research, including randomized clinical trials observational studies using questionnaires and registries. An analysis was conducted to compare the total number of negative factors between groups as well as to determine if any of these errors were present in a significant number of unsuccessful trials compared to the successful trials.

## Results

The analysis included a sample of 23 trials actively enrolling subjects in the previous calendar year. The successful trials (n = 7) averaged two identified issues per trial, whereas the unsuccessful trials (n = 16) averaged four identified issues per trial. Trials that were behind on enrollment had twice as many common issues as those that remained on track with recruitment goals.

**Figure 1:**
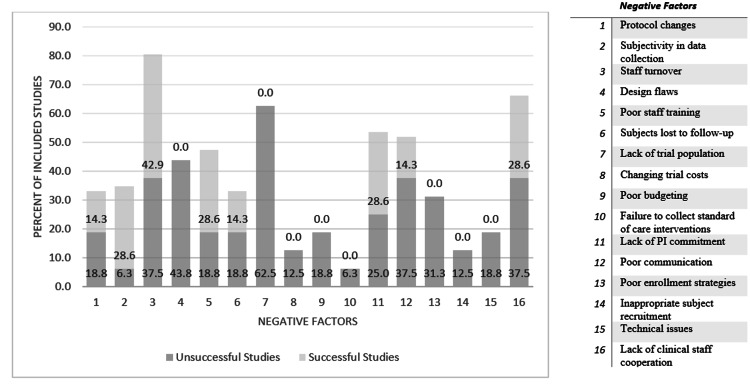
Percent of studies in each group, successful and unsuccessful, with each negative factor PI: Principal investigator

Staff turnover, lack of cooperation of clinical staff (e.g., floor nursing staff, operating room staff, and other staff not specifically involved with the trial), lack of trial population, lack of PI commitment, and poor communication occurred most frequently in the sample overall (Figure 1). We analyzed individual factor occurrence within each group to compare which issues were overcome in trials that continued to reach enrollment goals and which issues were common in failing trials.

Of the 16 common issues, seven were completely absent in successful trials: (1) lack of a trial-eligible population, (2) design flaws, (3) poor enrollment strategies, (4) poor budgeting, (5) inappropriate subject recruitment, (6) changing trial costs, and (7) standard-of-care interventions not considered. It is worth noting that even the successful trial group did not escape factors that may have hindered or slowed success, with staff turnover being the most common factor. While these factors were still observed in successful trials, they were observed with less frequency than in unsuccessful trials (Table 2).

**Table 2:** Most common difficulties observed in successful trials

Factor	Frequency (n = 7)
Staff turnover	3 (42.9%)
Lack of principal investigator commitment	2 (28.6%)
Poor staff training	2 (28.6%)
Subjectivity in data collection	2 (28.6%)
Lack of clinical staff cooperation	2 (28.6%)

The top five issues found in the 16 unsuccessful trials are shown in Table 3. The two most common issues in unsuccessful studies-lack of a trial-eligible population and design flaws-were completely absent in all sampled successful research.

**Table 3:** Most common difficulties observed in failed trials

Factor	Frequency (n = 16)
Lack of trial population	10 (62.5%)
Design flaws	7 (43.8%)
Poor communication	6 (37.5%)
Staff turnover	6 (37.5%)
Lack of clinical staff cooperation	6 (37.5%)

Of the 16 identified issues, the two shared by both trial groups were staff turnover and lack of clinical staff cooperation.

## Discussion

There is no single definitive cause for trial failure; rather, it is the combination of negative factors that hinders a project’s success. As discussed by Fogel, “each of the facets of protocol design, execution, and successive trial planning offers opportunities for trading off different concerns as well as simply making inappropriate judgments leading to poor outcomes” [4]. Specific attention should be given to the seven identified issues that did not occur in any successful study in our review: (1) lack of a trial-eligible population, (2) design flaws, (3) poor enrollment strategies, (4) poor budgeting, (5) inappropriate subject recruitment, (6) changing trial costs, and (7) standard-of-care interventions not considered.

The most prevalent issues in successful studies pertained to staffing (Table 2). Frequent staff turnover, subjective data collection, and issues with commitment, training, and communication are problems for many academic medical centers; they require both departmental and institutional commitments to resolve but can ultimately be overcome [5]. To remain on track with enrollment goals, departments must proactively tend to issue not only of staff turnover but also of lack of PI commitment.

In contrast, commonly identified issues in unsuccessful studies are difficult to control once a trial is launched. Early planning, including consideration of study methods and outcomes and feasibility analysis, can help prevent trials from flatlining. For example, the lack of a trial population should be determined early in the process. Does the site have access to the population being studied? What is a realistic enrollment goal? Do the inclusion and exclusion criteria narrow the scope too much? Can those criteria be loosened and still address the study question appropriately?

Furthermore, investigators must go beyond the availability of subjects to also consider the burden a trial may place on a subject; interventions perceived as too costly, invasive, or lengthy may impact a subject’s willingness to participate and lead to overestimating the quantity of eligible subjects with a limited overall reach of the trial. When the study begins enrollment, these issues may make it difficult to remain on track with recruitment goals.

Additionally, because some subjects who enroll in clinical research studies receive medical treatments, researchers must consider and plan for standard-of-care treatments or interventions that may influence study variables. For example, a researcher recording the effect of drug dosage on treatment groups in the protocol should consider underlying conditions, concomitant medications, and treatments that could impact the effect measurements. Such design flaws may result in underestimating the budget needed to reach trial completion. Fluctuating study costs and poor budgeting may contribute to a study’s inability to reach enrollment goals.

Design flaws are a major issue. Having a statistical consultation before finalizing the protocol can help ensure study procedures properly evaluate the intended aim. Adaptive designs can be used to improve a study, but they require the requisite expertise. Each study should be undertaken using project management principles, including a communication plan that includes a collective understanding of standard operating procedures, roles, desired metrics and timelines, and the orientation of study and hospital staff. An effective communication plan addresses issues with the study’s aims, personnel, methods, timeline, and purpose. Including stakeholders in the planning of the study early on and during the course of the study can bridge the “poor communication” gap and improve clinical staff cooperation. Keeping these issues in mind in the planning phase of a trial can help minimize their occurrence, which is critical to a trial’s success.

When a trial fails to reach its enrollment goals, neither positive nor negative results can be recorded, and the study subjects may have been put at risk for no benefit. Without a noted conclusion, data collected by a trial cannot be disseminated through publication to the medical community and the public at large. It is important to inform the medical community of a trial’s results, regardless of the outcome. However, when studies fall short of enrollment goals, any partially collected data are rendered useless. Completing a study and producing a publication reduces the chance of biased or repeated work, which limits the potential wasted resources used by trials from inception to translation. This is especially important because no research idea is necessarily exclusive to one investigator, and similar work may be performed across multiple institutions [6].

Limitations to our study include its retrospective nature. As this study was limited to trials that required coordinator support through the Office of Research in the Department of Anesthesiology at the University of Florida College of Medicine, all PIs were affiliated with that department, and the possibility of selection bias should be considered. Consideration should also be given to the heterogeneity of the trials reviewed; although trial designs and interventions varied in our sample size, we focused on trials with coordinator involvement. Data availability and quality were other limitations, as our review was contingent on the quality of the primary sources. Finally, our research has identified a significant knowledge gap regarding clinical trial failure, which has limited our ability to gather relevant and useful background data. Nevertheless, we feel our review offers valuable lessons about factors that contribute to clinical trial failure. In the future, these results could be broadened by including trials that do not include a clinical coordinator for study support and are instead coordinated by faculty, fellows, and residents, as well as trials led by PIs from additional specialties.

## Conclusions

These results can be used to guide research trial planning with the goal of reducing common issues before trial activity. A planning process that enables a committee of experts to assess feasibility and evaluate resource allocation before enrollment can eliminate many common issues identified in unsuccessful studies. Keeping these issues in mind, members of clinical, statistical, and financial offices can ensure that a project is well vetted before using departmental time and resources.

Results from this project were presented as an oral presentation at the University of Florida College of Medicine’s Department of Anesthesiology 2018 Celebration of Research conference and have guided the development of a new research trial submission process. This process requires each project to be reviewed by a board of clinicians, statisticians, research professionals, and departmental leaders. These professionals will provide input from their perspectives on new trials moving forward, with the goal of anticipating and providing solutions to study issues before the project becomes active. Future analysis will be conducted after this process has taken effect to analyze its effectiveness in minimizing common issues associated with clinical research.
